# Supplementary material for: The phospholipid PI(3,4)P2 is an apical identity determinant
Source: Nat Commun. 2018 Nov 28;9:5041. doi: 10.1038/s41467-018-07464-8 (PMC6262019; doi:10.1038/s41467-018-07464-8)
Supplement: Supplementary file 1 — Supplemental Information [file 41467_2018_7464_MOESM1_ESM.pdf]

The phospholipid $PI(3,4)P_2$ is an apical identity determinant

Roman-Fernandez, Roignot *et al.*

SUPPLEMENTARY INFORMATION.

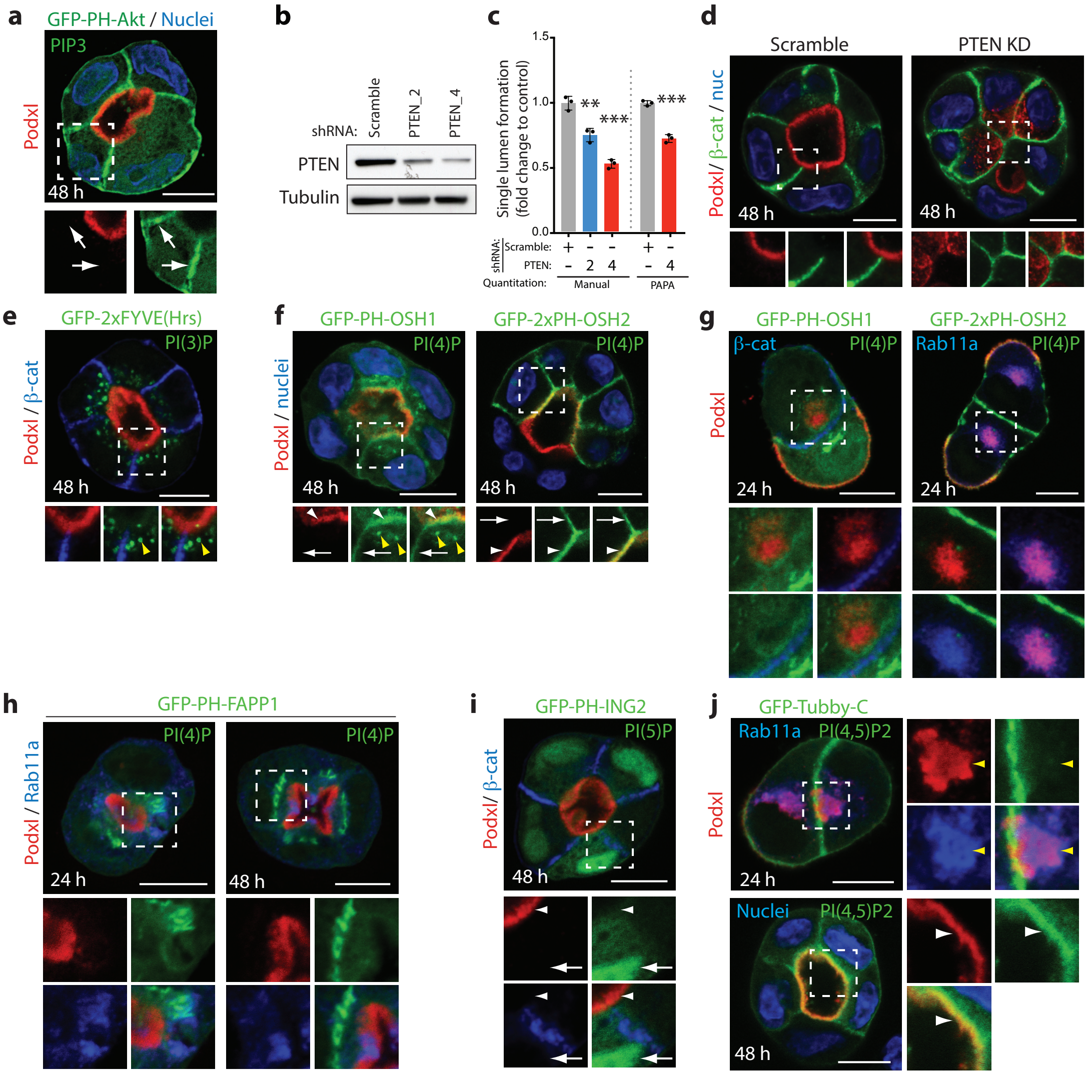

Supplementary Figure 1. PIP distribution and modifying enzyme expression, related to Figure 1.

(a) PIP_3 [GFP-PH-Akt] basolateral localization (white arrows) in 48h cysts stained for Podxl (red) and nuclei (blue).

(b-d) Effect of PTEN depletion on cyst polarization. (b) Total cell lysates of cells expressing control (scramble) or 2 different PTEN shRNAs, western-blotted for PTEN and tubulin. (c) Manual quantitation of cysts for single lumen formation for above condition, compared to automated quantitation with PAPA (see Supplementary Figure 3). Values are presented as a fold-change over control. Both manual and PAPA analysis: 3 replicates, ≥ 300 analysed cysts. *P* value (Student's t-

test): **$P \leq 0.005$, ***$P \leq 0.0005$. (d) Control (scramble) or PTEN-depleted cysts were labeled for Podxl (red), β-catenin (green) and nuclei (blue). Note subcortical accumulation of Podxl vesicles upon PTEN KD. In all instances, cysts phenotypes are quantified 48h after plating.

(e) PI(3)P [GFP-2xFYVE(Hrs)] (green, yellow arrowheads) localization in 48H MDCK cysts stained with Podxl (red) and β-catenin (blue).

(f) PI(4)P localization in cysts at 48h stained with Podxl (red) and nuclei (blue), using the alternate probes GFP-PH-OSH1 (left panel) and GFP-PH-OSH2 (right panel) (both green). Note basolateral (arrow) PI(4)P, and apical PI(4)P localization overlapping with Podxl (white arrowheads), and punctate PI(4)P-positive/Podxl-negative (yellow arrowheads) vesicles.

(g) PI(4)P localization in cysts at the vesicular Podxl stage stained with Podxl (red) and either β-catenin (left panel) or Rab11a (right panel) (both blue), using the alternate probes GFP-PH-OSH1 (left panel) and GFP-PH-OSH2 (right panel) [both green].

(h) PI(4)P [GFP-PH-FAPP1] localization in 24h and 48h cysts stained with Podxl (red) and Rab11a (blue).

(i) PI(5)P [GFP-PH-ING2] localization in 48h cysts stained with Podxl (red) and β-catenin (blue). Note nuclear PI(5)P (arrows), and lack of apical PI(5)P (arrowheads).

(j) PI(4,5)P_2 [GFP-Tubby-C] localization in cysts at the indicated stage stained for Podxl (red) and either Rab11a (top) or nuclei (bottom) (both blue). Note co-localization between Rab11a and Podxl vesicles (yellow arrowheads), but not GFP-Tubby-C. White arrowhead, apical PI(4,5)P_2.

All scale bars, 10 µm.

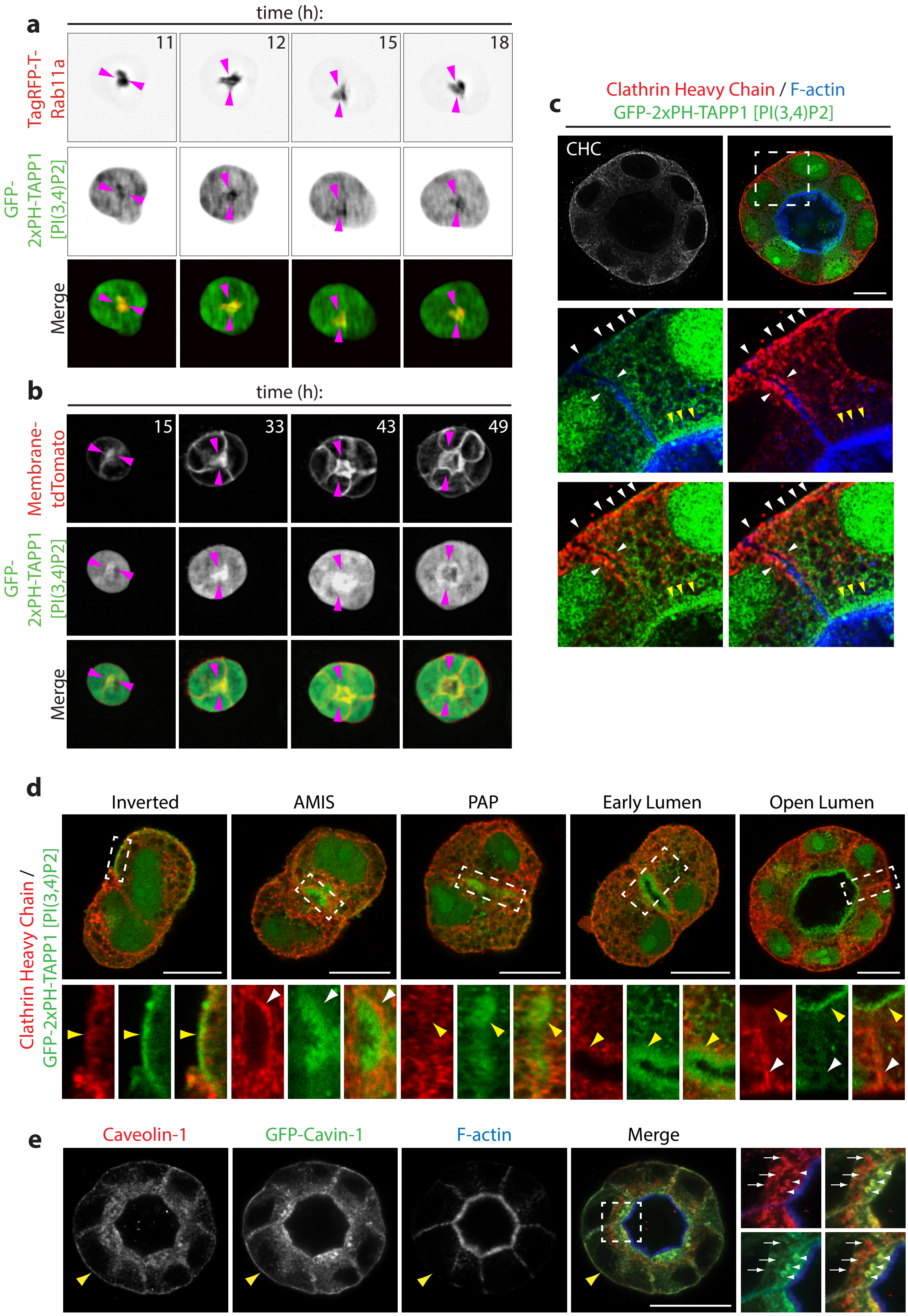

a
time (h):
11
12
15
18
TagRFP-T-Rab11a
GFP-2xPH-TAPP1 [PI(3,4)P2]
Merge
b
time (h):
15
33
43
49
Membrane-tdTomato
GFP-2xPH-TAPP1 [PI(3,4)P2]
Merge
c
Clathrin Heavy Chain / F-actin
GFP-2xPH-TAPP1 [PI(3,4)P2]
CHC
d
Inverted
AMIS
PAP
Early Lumen
Open Lumen
Clathrin Heavy Chain / GFP-2xPH-TAPP1 [PI(3,4)P2]
e
Caveolin-1
GFP-Cavin-1
F-actin
Merge

Supplementary Figure 2. Analysis of $PI(3,4)P_2$ localization, related to Figure 3.

(a,b) Time-lapse dual color confocal imaging of cysts expressing EGFP-2xPH-TAPP1 ($PI(3,4)P_2$) and either (a) TagRFP-T-Rab11a or (b) Membrane-tdTomato. Images were taken every hour from (a) ~8-24h (b) ~8-52h after plating. Stills are presented from (a) 11-18h or (b) 15-49h. Magenta arrowheads, luminal.

(c,d) Clathrin Heavy Chain (CHC, red) and $PI(3,4)P_2$ [GFP-2xPH-TAPP1] localization in open lumen cysts (c) and during MDCK cyst formation (d). Yellow arrowheads, luminal or peripheral. White arrowheads, CHC localization.

(e) Caveolin-1 (red), GFP-Cavin-1 (green) and F-actin (blue) in cysts. White arrowheads, dual Caveolin-1/GFP-Cavin-1 vesicles; White arrows, Cavin-1-negative Caveolin-1-positive vesicles. Yellow arrowheads, basal localization of Cavin-1/Caveolin-1.

All scale bars, 10 µm.

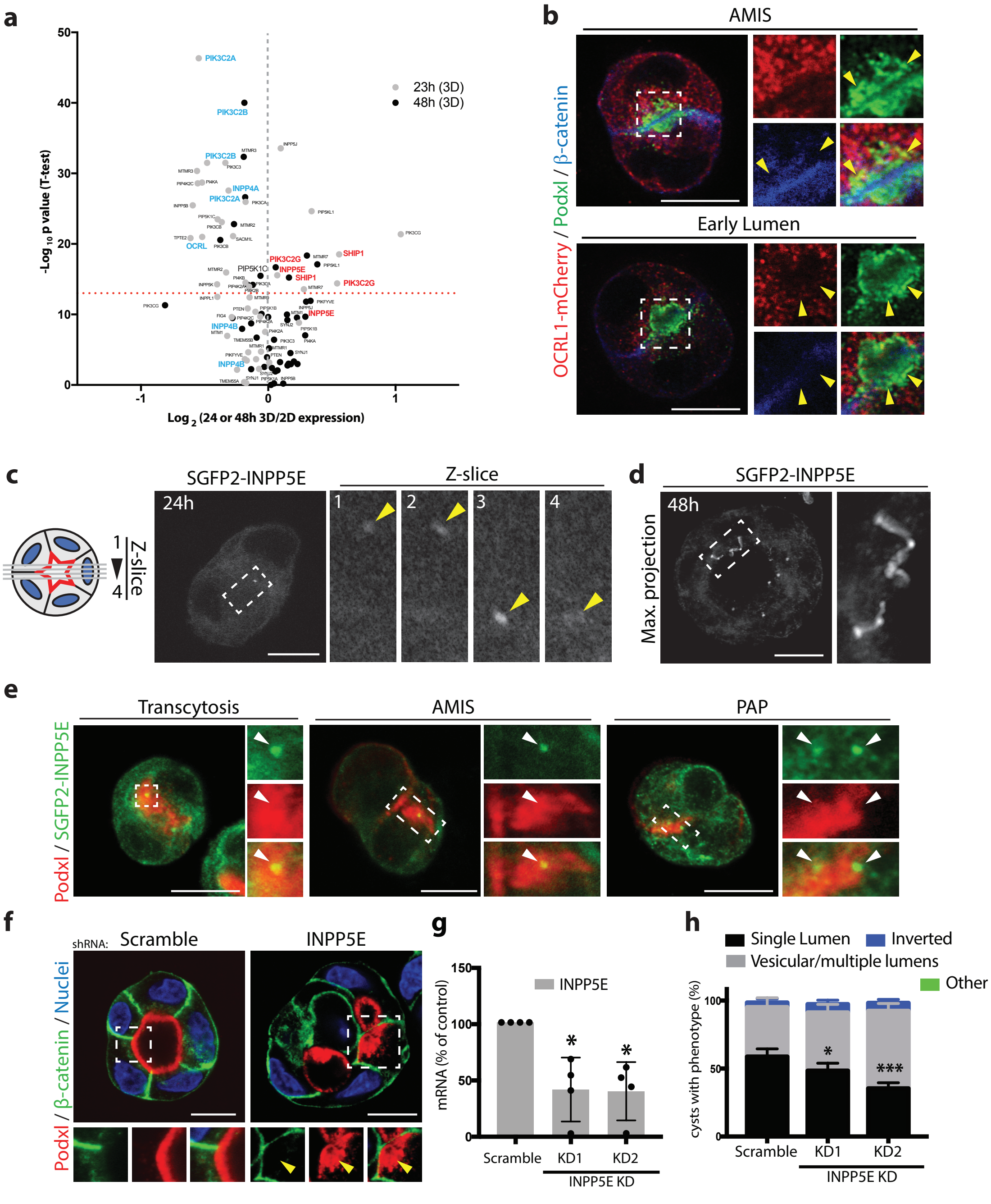

Supplementary Figure 3. INPP5E contributes to apical domain formation, related to Figure 4.

(a) Scatter plot comparing PIP-modifying gene expression by qPCR at 23h (grey dots) or 48h (black dots) (X axis, Log2 expression) to experimental replicate significance (Y axis, -Log10). Expression is normalized as fold change compared to 2D expression levels. Red dashed line, *P* value (Student's t-test), p=0.05. Names shown in bold are increased (red) or decreased (blue) potential genes of interest for PI(3,4)P_2 regulation.

(b) OCRL1-mCherry (red) localization at AMIS and early lumen stages in cysts stained with Podxl (green, yellow arrowheads) and β-catenin (blue).

(c) SGFP-INPP5E localization at 24h. Right, higher magnification of the boxed region, in sequential Z planes of the medial cyst region, taken at 1.08 uM intervals. Yellow arrowheads, INPP5E.

(d) Maximum intensity projection of a confocal stack of SGFP-INPP5E localization images from a cyst at 48h. Right, higher magnification of boxed region.

(e) SGFP-INPP5E (green) localization at vesicular, AMIS and PAP stages. Cysts are stained with Podxl (red). White arrowheads, INPP5E.

(f) Control (scramble) or INPP5E-depleted cysts stained with Podxl (red), β-catenin (green) and nuclei (blue). Yellow arrowhead, vesicular Podxl.

(g) *INPP5E* expression levels in control and INPP5E-depleted cells quantified by RT-qPCR analysis. Mean +/- s.d., 4 experiments/1 well per experiment. *P* value (Student's t-test): **P*≤ 0.05

(h) Quantitation of cysts phenotypes upon control (scramble) or INPP5E KD using two independent shRNAs. Mean ± s.d., n ≥300 cysts assessed from 3 wells/condition/experiment, 5 independent experiments. *P* values (2-way ANOVA): **P*≤ 0.05, ****P*≤0.0001.

All scale bars, 10 μm.

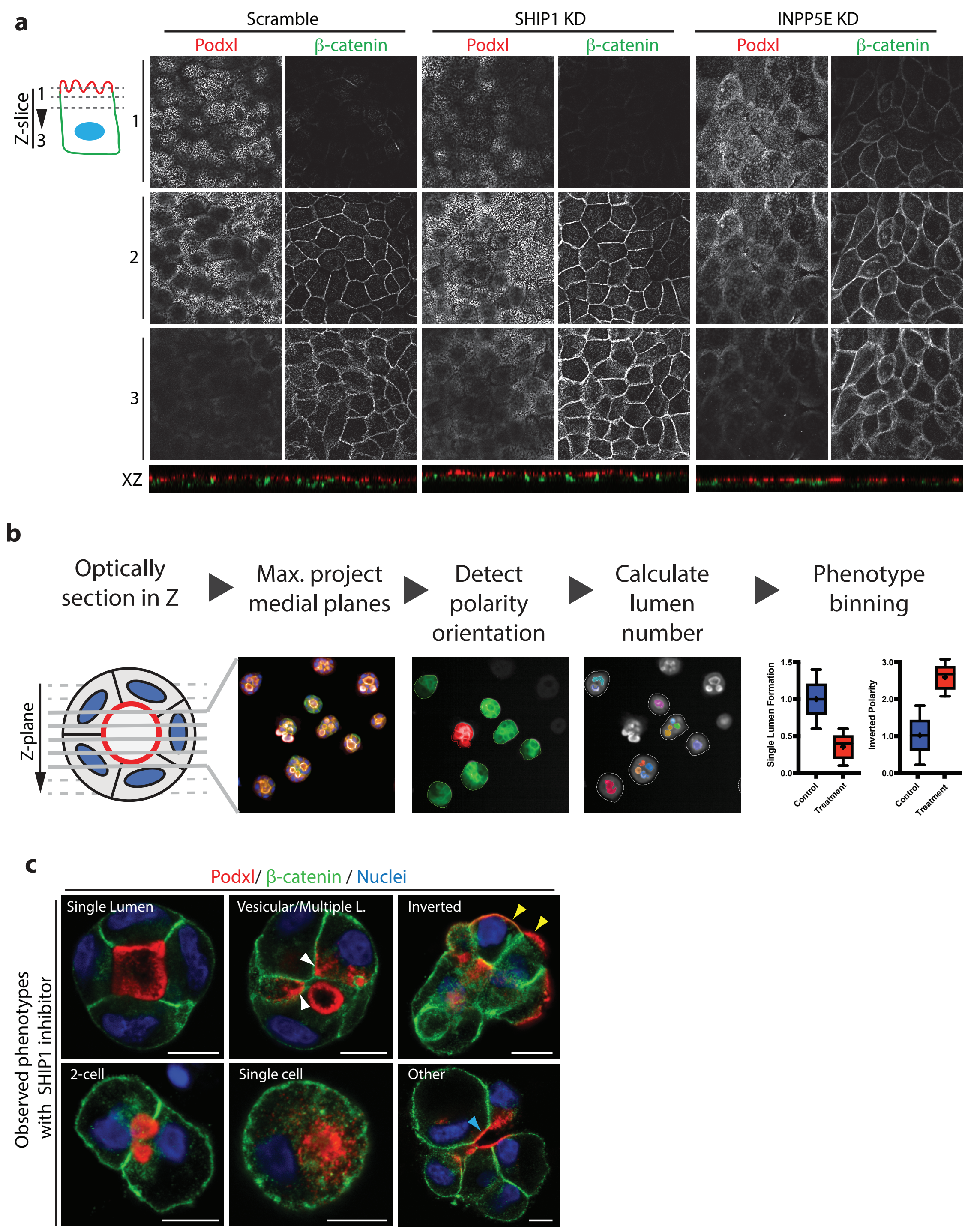

Supplementary Figure 4. Additional characterization of PIP-modifying enzymes, related to Figure 4.

(a) (Left) Schematic representation of a polarized, filter-grown epithelial cell. Dashed lines represent the confocal *xy* section, as indicated. (Right) Podxl or β-catenin staining in filter-grown, polarized MDCK monolayers stably expressing either scramble, SHIP1 or INPP5E shRNAs. (Bottom) XZ projections of confocal slices show apical-basal polarization of monolayers.

(b) Schematic representation of a Pipeline for semi-Automated Polarity Analysis, PAPA. MDCK cysts were cultured in 3D for 24-72h then fixed and stained with Podxl to mark the apical domain, Phalloidin to mark the cortex, Whole Cell Stain to mark cytoplasm and Hoescht to mark the nucleus. Multiple confocal optical sections of MDCK cysts were imaged. A maximum projection of the medial planes was applied, followed by analysis of the resultant images, which includes machine learning to detect polarity orientation based on the localization and intensity of apical markers and the detection and quantification of number of lumens per cyst. Final statistical analysis was performed to calculate percentages of polarity phenotypes, normalized to control, with an example plot presented.

(c) Control or SHIP1-inhibited cysts stained with Podxl (red), β-catenin (green) and nuclei (blue). White arrowheads, vesicular Podxl; yellow arrowheads, peripheral Podxl; blue arrowheads, disruption of cell-cell adhesions. Scale bars, 10 μm.

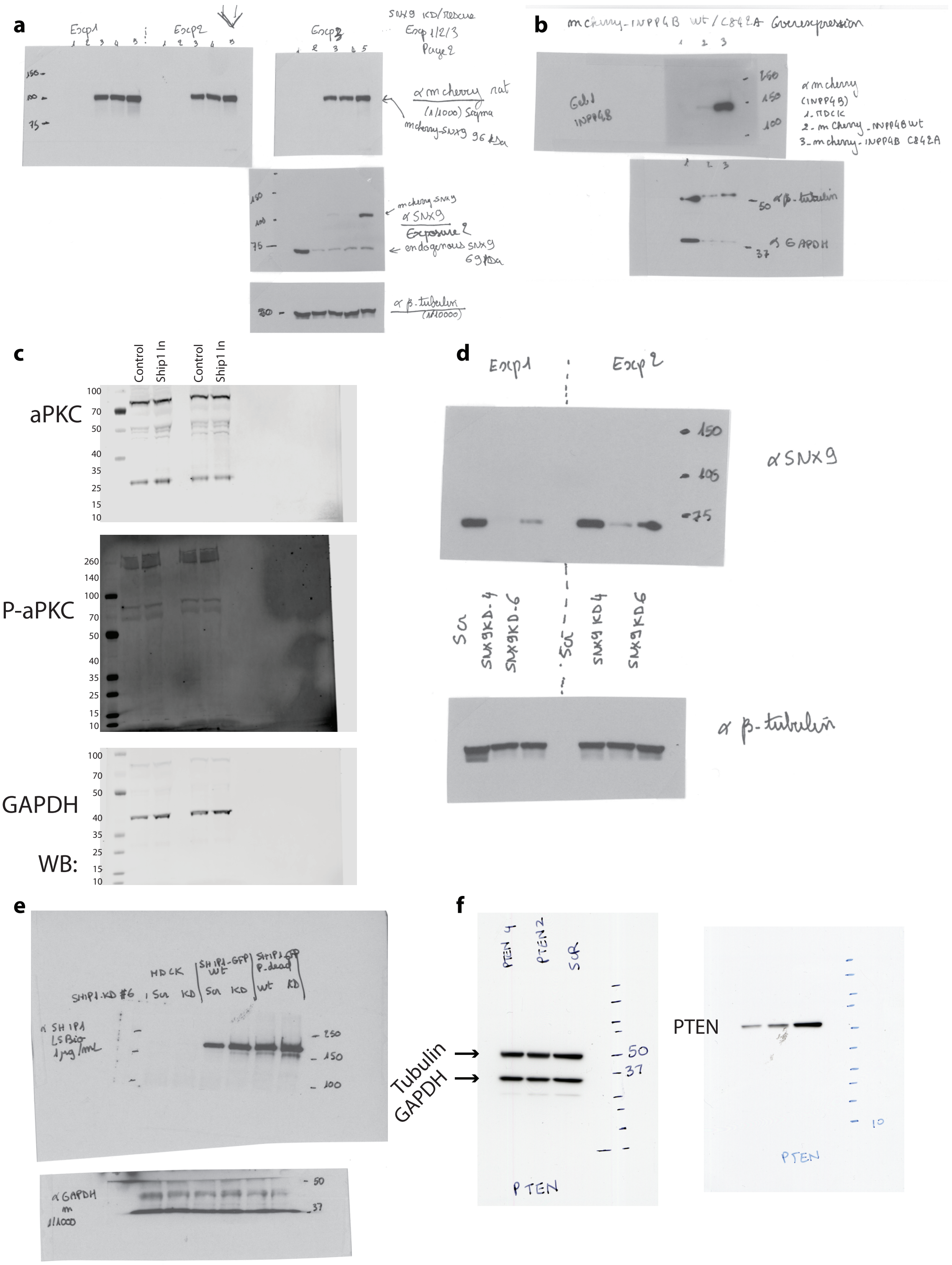

Supplementary Figure 5. Uncropped Western blots from main figures.

Uncropped membranes related to (a) Figure 9f; (b) Figure 7a; (c) Figure 8a; (d) Figure 9c; (e) Figure 4f and (f) Supplementary Figure 1.

Supplementary Table 1. Primary antibodies

Protein	Source	Catalogue #	Species	Dilution(s)
Acetylated Tubulin	Sigma	T-6793	Mouse	IF 1:1000
aPKC (C-20)	Santa Cruz	sc-216	Rabbit	IF 1:200
β-catenin	Santa Cruz	sc-7199	Rabbit	IF 1:200
Caveolin-1	BD Biosciences	610060	Rabbit	IF 1:500
Cherry	Life Technologies	M11217	Rat	WB 1:500
Clathrin Heavy Chain (X22)	F. Brodsky		Mouse	IF 1:1000
GAPDH	Millipore	MAB374	Mouse	WB 1:20,000
GAPDH	Cell Signaling	14C10	Rabbit	
GFP	Roche	11814460001	Mouse	WB 1:3000
GFP	Life Technologies	A-11122	Rabbit	WB 1:1,000; IP 1:500
Gp135/Podxl	G. Ojakian		Mouse	IF 1:1,000; WB 1:5,000
HA (Y-11)	Santa Cruz	sc-805	Rabbit	WB 1:1000
Myc (9B11)	Cell Signaling	2276	Mouse	WB 1:1000
Par3	Millipore	07-330	Rabbit	IF 1:100
PI(3,4)P2	Echelon	Z-P034	Mouse	IF: 1:100
Rab11a	Invitrogen	71-5300	Rabbit	IF 1:500
RFP	Invitrogen	R10367	Rabbit	WB 1:1000
SHIP1	LSBio	LS-C145498	Rabbit	IF: 1μg/ml
SNX9	GeneTex	GTX83599	Mouse	IF: 1:100

Supplementary Table 2. RNAi target sequences

Target Gene	RNAi Sequence (5' – 3')
INPP4A_5	GCTGCCAGTCCATAATCTACA
INPP4B_2	GCTCATGACACCGTTCAAACC
INPP5E_1	AAGGTGAGCGAGAGGCTGCTG
INPP5E_2	AAAGGGTCCATCTTCAAGGGC
PIK3C2A_5	GCTCAGATATCCAGCAATAAT
PIK3C2B_1	GGACAGGAAGCCTTGTGAGAA
PIK3C2G_1	CCTGGAAGCAACAAGTCATTT
PTEN_2	AATCCACCACAGCTAGAACTT
PTEN_4	AAACCCGGAGGCTAGCAGTTC
Scramble	CCGCAGGTATGCACGCGT
SHIP1_1	AAGGATCTCTATGGCGAAGTC
SHIP1_2	AAAGACGCGGGATGATTCTGC
SNX9_1	AATCCCTTCTCTTCCAGATAA
SNX9_2	AAGCGGGTTGGCACTATGTCT

Supplementary Table 3. RT-qPCR primer list

Target	Primer	Sequence (5' – 3')
FIG4	Fwd	ACCGCACTAACACAGCACAG
	Rev	CTGGGTCCATGGTGCTATCT
GAPDH	Fwd	AGTCAAGGCTGAGAACGGGAAACT
	Rev	CATGGTTCACGCCCATCACAAACA
INPP4A	Fwd	CCATCGCTAGATCGAAAACC
	Rev	TCTCCGTCTGTGCATGTTTC
INPP4B	Fwd	ATGAGCACCAGTTGCACAAG
	Rev	ACATTCTCTATGCGGCATCC
INPP4B(2)	Fwd	CGGTATTCGTTTCACCTGTTGCA
	Rev	GCACTGCTCAAGAGTCACTGAC
INPP5D (SHIP1)	Fwd	CCTCTGGAACATCCGAATTG
	Rev	ATGCCGGTCTTCACGTTATC
INPP5E	Fwd	AAGCTAAGGCAAAGCCCTTC
	Rev	GCAGGGAGGTACTCAAGCAG
INPP5J	Fwd	TGACCTCGTGTTCTGGTTTG
	Rev	GCAAACTTGACGAAGTGCAG
INPP5K	Fwd	AGCACTTTGACCGGATCTTG
	Rev	AAATGCAGCCCAAAGTCCTC
INPPL1	Fwd	ATGTGCCTTCATGGTGTGAC
	Rev	ACCTCAAATGTCCCGAACAC
MTM1	Fwd	TCCATATCGTGCCTCAGATG
	Rev	TGAATCCATGACAGCACTGG
MTMR1	Fwd	GTGGGTCCCAATGATAAACG
	Rev	CGGGCATCGAAGATGATAAG
MTMR2	Fwd	GTGGAAAGCGAAGCAAAGAG
	Rev	GCAACAGCATTGACACTTGG
MTMR3	Fwd	TGTTTCTGCAGTGGCTTGAC
	Rev	TCCAAACAGGCAGGAATAGG
MTMR7	Fwd	TTCCCTGTGCCTTTGAGTTC
	Rev	GTTCTTGTCGCTCCTTTTGG
MTMR9	Fwd	CTGGCATTCTTTCCTTCCTG
	Rev	GAAGGGCAGACAGCAAATTC
MTMR14	Fwd	TCCCTGATTTCCTGACTTGC
	Rev	TGTGTTTGTTGCACCAGGTC

OCRL	Fwd	TGGACCGAGGCAAAGATTAC
	Rev	ATGAGTTTGGTGACGGGAAC
PI4K2A	Fwd	GCGTTTTGAAGCAGAACCTC
	Rev	TTGCGGATGATGTAGTCCAG
PI4K2B	Fwd	CAGCTGGTACAAATGCCTTG
	Rev	GGAGGAAAAGAATGGCTTCC
PI4KA	Fwd	CAACCCTGAATGTCATGTCG
	Rev	CCTGCAGATGTTGTCAATGG
PI4KB	Fwd	ACTGCAGTCCATTTGGGAAC
	Rev	TGCCACTGTCAGCAGAAATC
PIK3C2A	Fwd	TGCTGGATGACAGTTTCGAG
	Rev	GCTGAAAATGAAGGGCTCAG
PIK3C2B	Fwd	GTCATTTTCCGCTGCTTCTC
	Rev	TTGTGTTTCTGCAGCCAGTC
PIK3C2G	Fwd	TACATGCAGGATTGCCTGAG
	Rev	TTGTTGCTTCCAGGTCTGTG
PIK3C3	Fwd	TTCCTCCTCCAATGAAGCTG
	Rev	ACTGTTTGCGGAACTCTTGG
PIK3CA	Fwd	GCCTCCAATCAAACCTGAAC
	Rev	GAACAGCAAAACCTCGAACC
PIK3CB	Fwd	CACCAGAACATGAACCATCG
	Rev	ACAGCCACAATGAGCTTTCC
PIK3CD	Fwd	TGGAATTCTGGACCAAGGAG
	Rev	TCTTGATGGTGCTGAGGTTG
PIK3CG	Fwd	CAACAACTGCGTCTTCATCG
	Rev	CCTTCTTCTTGGCCATCTTG
PIKFYVE	Fwd	AACAAGCCATGGAGAGGTTG
	Rev	AATGATGTCCCTCCAAGACG
PIP4K2A	Fwd	TGCCCAGTTAAAGCTCATGG
	Rev	TTCCCCGTCATTCTCTTCAC
PIP4K2B	Fwd	TCACCAGGAATGTGTTCAGC
	Rev	TGCCCTTCATTGAGGAAGTC
PIP4K2C	Fwd	TCGAGTTAGCGTGGACAATG
	Rev	TTCCTTATCGCTGGCTTCTC
PIP5K1A	Fwd	TACCAAACCAGAGCGTGATG
	Rev	CGAAAGTAACGGAAGGCAAC

PIP5K1B	Fwd	AGATGCCACACCTGGAAAAC
	Rev	TGGCTTGGAAGTGAGATTCC
PIP5K1C	Fwd	TCATCGACATTCTGCAGTCC
	Rev	CGGTGTTGCTCATGAACTTG
PIP5KL1	Fwd	TCAAGGCAAGACCATTGACC
	Rev	TGTAATCGAGCACGTTGAGC
PTEN	Fwd	CAGTGGCGGAACTTGCAATC
	Rev	TCGTGTGGGTCCTGAATTGG
SACM1L	Fwd	CAACCATGTCCTGAATGTGG
	Rev	TGATCTGCCCTTTCCAAGAG
SNX9	Fwd	GTGGTAGCAGATCCCAGGAA
	Rev	GGCTGACCCAAACTTAACCA
SYNJ1	Fwd	TGTGCAGCCATCAAGAAGAC
	Rev	TGTGGGTGATGCCTTAGATG
SYNJ2	Fwd	TGCTGGTGACTTTTGCAGAC
	Rev	TTCGGATGATCTCCTCTTGC
TMEM55A	Fwd	TCAGGCATGACAAAGCAGAG
	Rev	TAACCACATGCTGGTGAAGC
TMEM55B	Fwd	TCATCTGTGGACACTGCAAG
	Rev	ATCTGCGCCCAATAGATGAC
TPTE2	Fwd	CATTGTAAGGGAGGCAAAGG
	Rev	TTATCTGTTCGCCGTTCTCC
