# Supplementary material for: The phospholipid PI(3,4)P2 is an apical identity determinant
Source: Nat Commun. 2018 Nov 28;9:5041. doi: 10.1038/s41467-018-07464-8 (PMC6262019; doi:10.1038/s41467-018-07464-8)
Supplement: Supplementary file 3 — Description of Additional Supplementary Files [file 41467_2018_7464_MOESM3_ESM.pdf]

Supplementary Movie 1. Time-lapse imaging of Rab11a and PI(3,4)P2 localization during cyst development, related to Supplementary Figure 2a. Time-lapse dual-color confocal imaging of cysts expressing TagRFPT-Rab11a (left, inverted greyscale; red in merge) and GFP-2xPH-TAPP1 (middle, inverted greyscale; green in merge), with images taken every 60 min from 16-52h after plating. Merge, right.

Supplementary Movie 2. Time-lapse imaging of apical domains and PI(3,4)P2 localization during cyst development, related to Supplementary Figure 2b. Time-lapse dual-color confocal imaging of cysts expressing Membrane-targeted Tandem-dimer Tomato (TdTomato; left, inverted greyscale; magenta in merge) and GFP-2xPH-TAPP1 (middle, inverted greyscale; green in merge), with images taken every 60 min from 16-52h after plating. Merge, right.
